# Quality of cost evaluations of physician continuous professional development: Systematic review of reporting and methods

**DOI:** 10.1007/s40037-022-00705-z

**Published:** 2022-03-31

**Authors:** David A. Cook, John M. Wilkinson, Jonathan Foo

**Affiliations:** 1 School of Continuous Professional Development, Mayo Clinic College of Medicine and Science, Rochester, MN USA; 2 Division of General Internal Medicine, Mayo Clinic, Rochester, MN USA; 3 Department of Family Medicine, Mayo Clinic, Rochester, MN USA; 4 School of Primary and Allied Health Care, Monash University, Victoria, Australia

**Keywords:** Education, medical, Education, continuing, Costs and cost analysis, Cost effectiveness

## Abstract

**Introduction:**

We sought to evaluate the reporting and methodological quality of cost evaluations of physician continuing professional development (CPD).

**Methods:**

We conducted a systematic review, searching MEDLINE, Embase, PsycInfo, and the Cochrane Database for studies comparing the cost of physician CPD (last update 23 April 2020). Two reviewers, working independently, screened all articles for inclusion. Two reviewers extracted information on reporting quality using the Consolidated Health Economic Evaluation Reporting Standards (CHEERS), and on methodological quality using the Medical Education Research Study Quality Instrument (MERSQI) and a published reference case.

**Results:**

Of 3338 potentially eligible studies, 62 were included. Operational definitions of methodological and reporting quality elements were iteratively revised. Articles reported mean (SD) 43% (20%) of CHEERS elements for the Title/Abstract, 56% (34%) for Introduction, 66% (19%) for Methods, 61% (17%) for Results, and 66% (30%) for Discussion, with overall reporting index 292 (83) (maximum 500). Valuation methods were reported infrequently (resource selection 10 of 62 [16%], resource quantitation 10 [16%], pricing 26 [42%]), as were descriptions/discussion of the physicians trained (42 [68%]), training setting (42 [68%]), training intervention (40 [65%]), sensitivity analyses of uncertainty (9 [15%]), and generalizability (30 [48%]). MERSQI scores ranged from 6.0 to 16.0 (mean 11.2 [2.4]). Changes over time in reporting index (initial 241 [105], final 321 [52]) and MERSQI scores (initial 9.8 [2.7], final 11.9 [1.9]) were not statistically significant (*p* ≥ 0.08).

**Discussion:**

Methods and reporting of HPE cost evaluations fall short of current standards. Gaps exist in the valuation, analysis, and contextualization of cost outcomes.

**Supplementary Information:**

The online version of this article (10.1007/s40037-022-00705-z) contains supplementary material, which is available to authorized users. This material includes the full search strategy, operational definitions of the CHEERS elements, and a list of all included studies with key information.

## Introduction

Information about costs is essential to making informed decisions in health professions education (HPE) [1–5]. Although the number of published cost evaluations in HPE seems to be rising [6, 7], concerns remain regarding the quality of such research [2, 7, 8].

Complete, transparent reporting is a crucial component of research [9–11]. Reporting facilitates several activities, including appraisal of methods, replication or application of study interventions, and data extraction for evidence synthesis. The abstract has unique, complementary roles in expediting literature searches and attracting potential readers to read the main text [12–14]. Reflecting on reporting quality is particularly important in emerging fields when an understanding of key reporting elements is maturing. The Consolidated Health Economic Evaluation Reporting Standards (CHEERS) statement [10] was created to promote complete reporting for economic (cost) evaluations. Empiric data on the type and prevalence of specific reporting deficiencies for cost evaluations in HPE could inspire solutions and enhance awareness among those who can help remedy such problems (educators, funding agencies, editors).

A review of 59 cost evaluations in simulation-based medical education [6] found that studies are infrequent and report only limited information about cost resources (“ingredients”), but did not examine reporting quality in detail. Another review [15] appraised reporting quality using the BMJ Guidelines [16] (a predecessor of CHEERS) for a strategic sample of 78 cost evaluations in HPE, and found deficiencies in several areas including participants, currency, resource quantitation and pricing, and analysis. However, this study included only 16 *“full economic evaluations (i.e., studies that compared two or more interventions and considered both cost and learning outcomes).”* Moreover, the BMJ Guidelines focus on reporting of study methods and results, while neglecting important elements in the title, abstract, introduction, and discussion. As such, the reporting quality of cost evaluations in HPE remains incompletely characterized.

Methodological quality is distinct from reporting quality. Published tools and frameworks for methodological appraisal are helpful when performing peer review, deciding to apply results locally, and conducting literature reviews. For example, the Medical Education Research Study Quality Instrument (MERSQI) was developed as a generic tool to appraise methods of any quantitative HPE research study [17, 18]. However, the MERSQI does not focus on methodological issues specific to cost evaluations. The two reviews noted above [6, 15] used the MERSQI to appraise study quality generally; both found suboptimal methods, and one proposed operational clarifications to enhance the MERSQI’s relevance to cost evaluation [15]. However, neither review characterized cost-specific methodological limitations in detail. We recently described a “reference case” defining key methodological considerations for cost evaluations in HPE [19], but this has not yet been empirically applied. Further trialing of various tools for appraising the methods of cost evaluations, with attention to operational clarifications, may facilitate future applications.

To address these knowledge gaps, we sought to answer: What is the reporting quality of published HPE cost evaluations as appraised using the CHEERS, and what are common methodological shortcomings? We did this using articles identified in an ongoing systematic review of physician continuing professional development (CPD) (i.e., a convenience sample).

## Methods

This systematic review was a pre-planned aim of a comprehensive protocol-driven literature review of economic outcomes of CPD. We used a subset of the articles identified in our recently published scoping review [19], analyzing new (unpublished) data. This review was conducted and reported in adherence to the Preferred Reporting Items for Systematic Reviews and Meta-Analyses [20] and standards for economic evaluations [10, 21, 22].

### Data sources and searches

We worked with an experienced reference librarian to create a search strategy (see e‑Box in Electronic Supplementary Material [ESM]) that included terms related to the population (e.g., “Physicians”), intervention (e.g., “Education, Continuing”), and outcomes (e.g., “Economics, Medical,” “Costs and Cost Analysis”). We searched MEDLINE, Embase, PsycInfo, and the Cochrane Database, from each database’s inception through April 23, 2020. We identified three additional studies from included article reference lists.

### Study selection

We included all original comparative studies that reported the cost of physician CPD, without restrictions (such as language or date). We defined *physician CPD* as

*Activities intended to promote or measure the clinical knowledge/skills of physicians in independent medical practice through a) courses or assessments delivered in any modality or venue, whether or not *[continuing medical education]* credit is awarded, or b) self-directed learning or self-assessment activities for which credit is awarded *[19].

As comparative studies, we included studies with two or more groups, single-group studies with pre- and post-intervention assessment, cross-sectional studies that simultaneously evaluated training cost and training consequences (outcomes), and economic modeling studies based on a specific, real (not hypothetical) training intervention.

Each article was screened for inclusion independently by two reviewers (the authors or coinvestigators CRS, SM, and BBT recognized in the Acknowledgements), who first reviewed the title and abstract and then reviewed the full text if needed (inter-reviewer reliability, kappa = 0.73). Reviewers resolved all disagreements by consensus.

### Data extraction and quality appraisal

Two reviewers (DAC, JF) independently reviewed each included study to extract information on reporting and methodological quality.

We coded reporting quality using the CHEERS statement [10], iteratively discussing and operationalizing each reporting element (see Tab. S1 in ESM for operational considerations). We extracted additional detailed information about reporting of abstracts [14] and research questions [11, 23].

We appraised methodological quality in three ways. First, we appraised methodological quality generally using the MERSQI [18], which comprises domains of study design, sampling, outcomes (type, objectivity, and validity), and statistical analyses. Building on operational definitions used in a previous systematic review [15], we revised the coding criteria to accommodate specific features of cost evaluations, as outlined in Tab. 1. Second, we appraised cost-specific methods by coding key methodological features of cost evaluations using the “reference case” domains defined in our scoping review [19], namely: perspective, comparison, description of intervention(s), non-economic measures of effectiveness, training costs, valuation of monetary amounts, analysis, sensitivity analyses, and discounting (see details in Results). Third, we rendered an overall subjective rating of methodological quality using a framework developed by Clune for a review of cost evaluations of education [24]. Clune classified the study methods as supporting results that are “Plausible” (strong quantitation and pricing, effectiveness outcomes, and comparison), “Substantial” (substantial data but serious flaws), “Minimal” (minimal data; list costs, allege affordability, claim feasibility), or “Rhetoric” (claims of cost-effectiveness without data).

**Table 1:** Methodological quality appraised using the Medical Education Research Study Quality Instrument (MERSQI): operational considerations for cost evaluations and prevalence

Domain: Item	Operational adjustments	Level	Prevalence *N* (%) (*N* = 62)
Study design	Added option for economic modeling studies (score 1.5)	1‑group post-only (1)	6 (10%)
		1‑group pre-post, or modeling (1.5)	20 (32%)
		2‑group non-randomized (2)	16 (26%)
		2‑group randomized (3)	20 (32%)
Sampling: No. of institutions studied	No change	1 (0.5)	54 (87%)
		2 (1)	1 (2%)
		>2 (1.5)	7 (11%)
Sampling: Response rate	For cost data: Data derived from large record sets unlikely to reflect bias (e.g., institutional electronic health record or regional claims database) count as high (score 1.5)	<50% or not specified (0.5)	24 (39%)
		50–74% (1)	7 (11%)
		≥75% or large record	31 (50%)
Type of data (data source)	For cost data: Details of resource quantitation (both data source and quantity [number of units, not just total cost]) count as high (score 3). Cost alone counts as low (score 1)	Self-reported data, or cost without resource quantitation (1)	8 (13%)
		Objective measurement, or cost with data source and quantity (3)	54 (87%)
Validation of evaluation instrument: Content	For cost data: *“The degree to which the cost estimation encompasses all aspects of the true cost, encompassing processes to both identify and measure cost”* [15]. Evidence could include use of a formal framework (e.g., the Ingredients Method) or the involvement of experts in planning, empiric identification and selection of relevant resources (e.g., time-motion studies or process mapping), and substantiation that a robust data source was used to select, quantitate, or price resources (e.g., detailed description of a computer database)	Reported (1)	8 (13%)
Validation of evaluation instrument: Internal structure	For cost data: *“The degree to which the cost estimate is reproducible if the same method is followed”* [15]. Evidence could include replicability of the valuation or analysis (e.g., robust examination of the uncertainty of input parameter estimates [sensitivity analysis], independent valuation of costs by two investigators [inter-rater reliability], or comparing cost estimates derived at two different time points [temporal stability])	Reported (1)	9 (15%)
Validation of evaluation instrument: Relations with other variables	For cost data: *“The degree to which the cost estimate relates to cost estimates formed using alternative approaches”* [15]. Evidence could include examining predicted associations among results obtained using alternative approaches to economic modeling (e.g., sensitivity analysis comparing different base assumptions, valuation methods, statistical models, or economic theories)	Reported (1)	1 (2%)
Data analysis: Appropriateness	For cost data: The following count as “appropriate” (score 1): cost effectiveness ratio, net benefit, or other similar analysis of cost data	Inappropriate for study design (0)	37 (60%)
		Appropriate (1)	25 (40%)
Data analysis: Complexity	For cost data: The following count as “beyond descriptive” (score 2): cost effectiveness ratio, net benefit, visual display of cost-effectiveness	Descriptive analysis only (1)	37 (60%)
		Beyond descriptive analysis (2)	25 (40%)
Outcomes	For cost outcomes: As per Foo, we distinguished education costs in a “test setting” or a “real setting,” namely: *“Test settings are those in which the context does not match how the intervention would be utilized in actual practice (e.g., a hypothetical program that was not actually implemented). Real settings are where the intervention is evaluated in a context similar to its anticipated utilization in practice (e.g., an evaluation of a program that is taught to real students)”* [15]. However, we assigned points differently than Foo: score 1.5 for cost of education in test setting, score 2 for cost of education in real setting, score 3 for health care costs. Outcomes estimated from previously published research (including health care costs and non-cost outcomes) also score 1.5	Knowledge, skills, or education costs in a “test” or hypothetical training setting, or estimated from literature (1.5)	1 (2%)
		Behaviors in practice or education costs in a “real” training setting (2)	25 (40%)
		Patient effects, including health care costs (3)	36 (58%)

For each item in a given study, the design feature (study design, outcome, evaluation instrument, etc.) that supported the highest level of coding was selected. For example, for a study reporting both cost and effectiveness (non-cost) outcomes, the outcome corresponding to the highest-scoring level was selected for coding (and as a result, in some cases the design features in the cost evaluation [i.e., the features coded in this review] are less than those reported in this table)

### Data synthesis and analysis

As has been done previously [23], we calculated a completeness of reporting index for each report section (title/abstract, introduction, methods, results, and discussion) reflecting the unweighted average percentage of elements reported. We summed the section-specific reporting indices to yield an overall reporting index (all sections, maximum 500) and a main text reporting index (all except title/abstract, maximum 400).

We calculated inter-rater agreement on quality codes using kappa. We used the Kruskall-Wallis or Wilcoxon rank sum test to explore differences in reporting quality and methodological quality over time (dividing articles into four groups of roughly equal size according to publication year), and for structured vs unstructured abstracts. We used Spearman’s rho to explore correlations among the various reporting quality and methodological quality scores. We used SAS 9.4 (SAS Institute, Cary, NC) for all analyses. Statistical significance was defined by a two-sided alpha of 0.05.

## Results

We identified 3338 potentially eligible studies, of which 62 met criteria for inclusion; see Fig. S1 in ESM. One study, originally published in French, was translated for data abstraction. Tab. S2 in ESM contains a full list of included studies. Twenty-eight studies were “full economic evaluations” (comparison of two groups using empiric outcomes of both education costs and effectiveness).

### Participants and CPD topics

As per inclusion criteria, all studies involved practicing physicians. Specialties included family, internal, or general medicine (49 studies), surgery (6 studies), and pediatrics (3 studies). Twenty-one studies additionally involved other health professionals, including nurses (10 studies), nurse practitioners/physician assistants (8 studies), and postgraduate physician trainees (5 studies). CPD topics included general medicine (35 studies), drug prescribing (11 studies), procedural skills (7 studies), and pediatrics (3 studies).

### Reporting quality overall: CHEERS elements

**Fig. 1:**
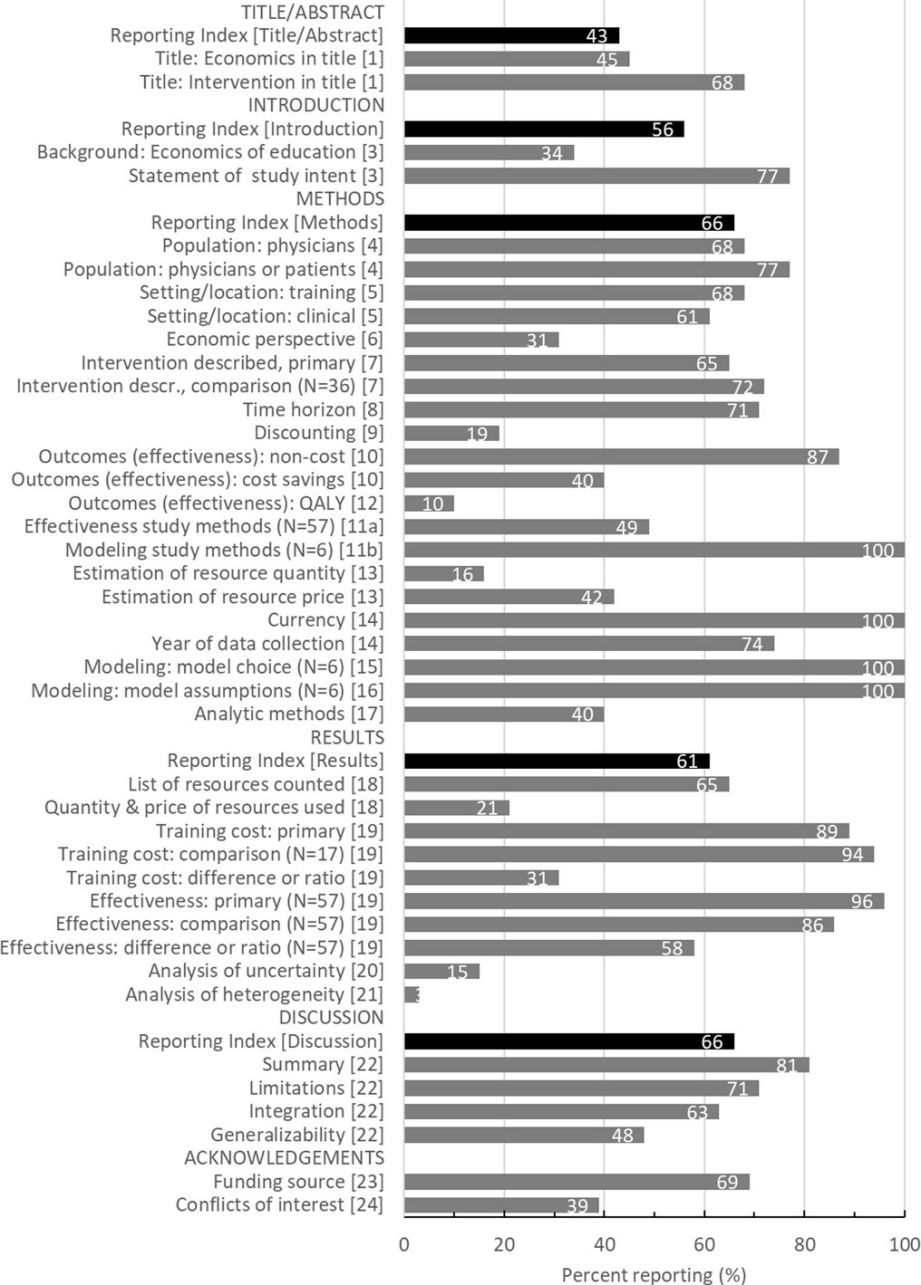
Reporting quality as per CHEERS guideline criteria. *N* = 62 except as indicated. Numbers in [brackets] indicate item number in CHEERS checklist [10]. Details on abstract reporting are provided in Fig. 2. Operational considerations used in coding are provided in Tab. S1 in ESM

The frequency of reporting of each CHEERS element is shown in Fig. 1. Inter-rater reliability was kappa ≥0.67 (“substantial” or “near-perfect”) for all these items. The reporting index (percentage of CHEERS elements reported in a given article section, maximum 100) ranged from 43 to 66 per section, namely: Title/Abstract mean (SD) 43 (20), Introduction 56 (34), Methods 66 (19), Results 61 (17), and Discussion 66 (30). The overall reporting index (sum of the five section-specific reporting indices, maximum 500) was 292 (83), ranging from 94 to 432 per study.

Only 28 studies (45%) mentioned economics or cost (or a similar term) in the title, and 21 (34%) mentioned the need to study the costs or economics of education in the Introduction. Forty-eight studies (77%) reported a specific statement of study intent (research question, purpose, goal, aim, or hypothesis). Among these, we further coded for four key elements of the statement of study intent, as defined in a previous review [11]: the population (reported in 33 [69% of 48]), the primary intervention (40 [83%]), the comparison or anticipated association (13 [27%]), and the outcome (44 [92%]). Thirty-eight (79%) reported a cost-related outcome in the statement of study intent, and 20 (42%) mentioned an effectiveness outcome.

The number of study participants was reported inconsistently: 42 studies (68%) reported the number of physicians trained and 23 (37%) reported the number of patients (i.e., in outcomes analyses); either physicians or patients were numbered in 48 (77%). The “intervention” training activity was well-described in 40 studies (65%). Among 36 studies with a separate comparison arm, the comparison activity was well-described in 26 (72%); this included 15 of 22 studies (68%) with no-intervention comparisons (i.e., explicit mention that the comparison group received no training) and 14 of 17 studies (82%) with an active training comparison. Given the critical role of context in cost evaluations, we coded both the training setting (physical location of training, experience/qualifications of CPD provider, or relationship between provider and physicians; reported in 42 [68%]) and the larger clinical context (patient demographics, or institution type, size, or infrastructure; reported in 38 [61%]).

The selection, quantitation, and pricing of resources (collectively, the valuation process) constitute the core of an economic analysis, yet these methods were reported infrequently. Although 40 (65%) listed the resources counted in valuation, only 10 (16%) reported the method for selecting these resources for inclusion. Likewise, methods of resource quantitation were reported infrequently: 10 studies (16%) reported both quantities and data sources, another 8 (13%) reported quantities without specifying the source, and 44 (71%) reported no quantities (i.e., reported the cost alone). The source of price information was mentioned in 26 (42%). Fifty-seven studies (92%) reported effectiveness (non-cost) outcomes; of these, 28 (49%) reported in detail the methods used to measure such outcomes.

As required for inclusion in this review, all studies reported the cost of at least one training intervention. However, only 13 (21%) detailed the quantities and prices of resources used. The CHEERS statement recommends reporting both the total costs for each intervention, and the incremental cost between interventions or relative to baseline (the cost difference or ratio). Fifty-five studies (89%) reported the total cost of at least one intervention, and 16 of 17 studies with an active comparison intervention (94%) reported the comparison’s total cost. Nineteen studies (31%) reported the incremental cost.

Although 44 studies (71%) reported at least a partial list of study limitations, only 30 (48%) discussed the generalizability of findings (i.e., applicability to other contexts). Forty-three (69%) listed the funding source, and 24 (39%) mentioned potential conflicts of interest.

### Reporting quality of abstracts

**Fig. 2:**
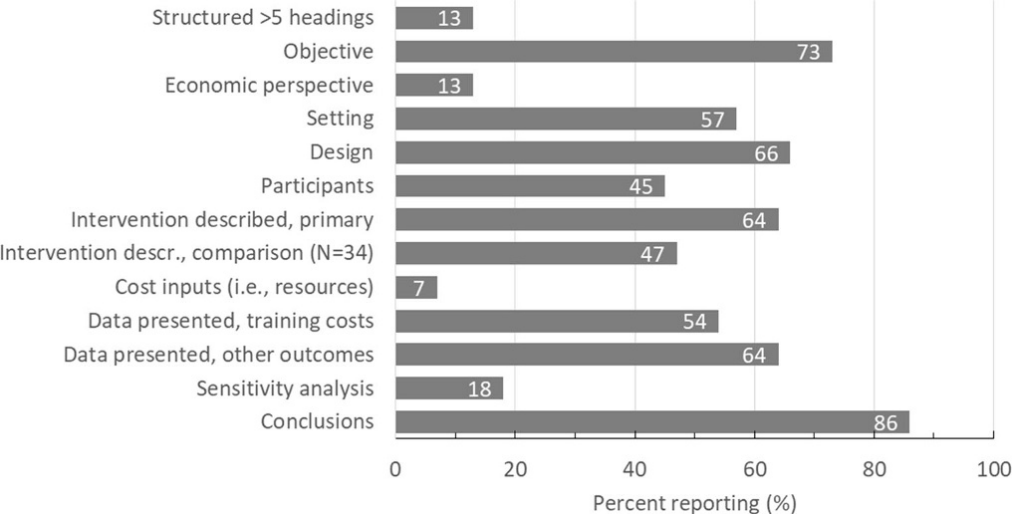
Reporting quality of abstract. *N* = 56 studies with abstract, except as indicated

The CHEERS guidelines recommend *“reporting a structured summary [abstract] of objectives, perspective, setting, methods (including study design and inputs), results (including base-case and uncertainty analyses), and conclusions”* [10]. We coded each abstract for these elements, and a description of participants and the primary and comparison training interventions (Fig. 2; Tab. S1 in ESM). Six studies (10%) had no abstract. Among the remaining 56, on average 5.8 (2.1) of 12 elements (median 6, range 2–10) were reported. Only 7 of 56 (13%) used a highly structured abstract (>5 headings), and none of these used headings specific to economic studies. Most (32 [57%]) used a four- or five-heading variation of purpose-methods-results-conclusions, and 17 (30%) were unstructured. The cost “inputs” (specific resources included in cost analyses, reported in 4 abstracts [7%]), economic perspective (7 [13%]), and sensitivity analyses (10 [18%], including 6 for training costs and 4 for clinical cost outcomes) were reported least often. Structured abstracts contained more elements than unstructured abstracts (mean [SD] 6.6 [1.7] vs 3.8 [1.7], *p* < 0.001), and highly structured abstracts contained more elements than less or unstructured abstracts (7.7 [2.1] vs 5.5 [2.0], *p* = 0.02).

### Methodological quality

We appraised general methodological quality with the MERSQI, using operational definitions summarized in Tab. 1. Scores ranged from 6.0 to 16.0, with mean (SD) 11.2 (2.4), median 11.0 (IQR, 9.5, 13.0). The domain with lowest scores was validation of the evaluation instrument: 50 studies (81%) reported zero evidence sources, and only 1 study (2%) reported all 3 of the sources appraised in the MERSQI (content, internal structure, and relations with other variables). Appropriate data analysis (operationalized as quantitation of resources) was also infrequent (25 [40%]).

We appraised cost-specific methodological quality according to the following “reference case” domains [19]:

- *Perspective*: 19 studies (31%) noted the economic perspective.
- *Comparison*: The reference case recommends comparison *“ideally be made with a plausible alternate active intervention”*; this was done in 17 of 62 studies (27%). Comparison with a no-intervention group (which typically offers less useful insights than an active comparison) was done in 22 studies (35%). Single-group designs, which *“contribute little to generalizable knowledge and are not preferred,”* were used in 26 studies (42%).
- *Description of intervention(s)*: Details were reported above.
- *Non-economic measures of effectiveness*: 56 studies (90%) reported effectiveness outcomes, including participation rates or satisfaction (18 [29%]), knowledge/skills (10 studies [16%]), physician behaviors (39 [63%]), and effects on patients (30 [48%]).
- *Training costs*: As per inclusion criteria, all studies reported training costs. The reference case recommends including *“time invested by faculty, support staff, and learners in all valuations of training cost.”* Time was reported for faculty, staff, learners, or any of these, in 21 (34%), 16 (26%), 16 (26%), and 29 (47%) studies, respectively.
- *Valuation*: Details were reported above for methods of selection, quantitation, and pricing. Additionally, 13 studies (21%) explicitly excluded potentially relevant resources such as startup costs, capital expenses, faculty or learner time, and donated materials.
- *Analysis*: The reference case recommends reporting combined *“expressions of cost and impact (e.g., cost-effectiveness ratio, net value, or benefit-cost ratio)”*; this was done in 25 (44%) of the 57 studies reporting effectiveness outcomes. Statistical comparisons of costs were performed in only 1 study (2%). Descriptive numeric analyses of costs, such as cost-effectiveness ratios and net benefits, were more common but still infrequent (17 [27%] and 9 [15%], respectively). Visual analyses (cost-effectiveness plane, decision acceptability curve) were employed in 2 studies (3%).
- *Sensitivity analyses*: 9 studies (15%) reported sensitivity analyses exploring uncertainty in training cost estimates (6 other studies reported sensitivity analyses for clinical cost outcomes). Probabilistic sensitivity methods were used in 4 studies (6%). Two studies (3%) conducted subgroup analyses exploring heterogeneity among training subgroups (e.g., across implementation sites).
- *Discounting*: 9 studies (15%) employed discounting in cost valuations, and 3 others (5%) explained why discounting was unnecessary in their study.

Finally, we rendered a subjective judgment of overall methodological quality (the strength of each study’s findings). We rated 6 studies (10%) as having “Plausible” results, 15 (24%) as “Substantial,” and 41 (66%) as “Minimal.” No studies were coded as “Rhetoric,” since such studies did not meet inclusion criteria. Scoring these as Plausible = 3, Substantial = 2, Minimal = 1, the mean (SD) was 1.4 (0.7).

### Changes over time

We divided studies by publication date into four groups of similar size. Reporting quality improved over time (see Tab. S3 in ESM), with the overall reporting index rising from mean (SD) 241 (105) in 16 studies before 1999 to 321 (52) in 17 studies after 2010; however, this trend did not reach statistical significance (*p* = 0.08). Methodological quality also varied over time, with total MERSQI scores rising from 9.8 (2.7) to 11.9 (1.9) and subjective methodological quality rising from 1.3 (0.6) to 1.7 (0.8); however, these changes likewise did not reach statistical significance (*p* ≥ 0.15). We also report differences in the frequency of reporting conflicts of interest (0/16 vs 12/17, *p* < 0.001), quantitation of resources (3/16 vs 4/17, *p* = 0.70), pricing of resources (4/16 vs 11/17, *p* = 0.09), and sensitivity analyses (1/16 vs 4/17, *p* = 0.61) over the same time periods (see Tab. S3 in ESM for details, including the interim chronological subgroups).

### Subgroup and correlational analyses

We found moderate to large, positive, statistically significant correlations between reporting and methodological quality, namely: overall reporting and MERSQI total score, rho = 0.62; overall reporting and subjective methodological quality, rho = 0.58; and MERSQI score and subjective quality, rho = 0.50 (all *p* < 0.001). We found a slightly lower correlation between abstract quality (number of items reported) and main text reporting (rho = 0.45, *p* < 0.001). Studies with structured abstracts had higher main text reporting (271 [47], maximum 400) than those with unstructured abstracts (229 [87]), *p* = 0.04.

## Discussion

In this systematic review of physician CPD, studies reported 58% of the items suggested in the CHEERS reporting guideline (overall reporting index 292/500). Areas of particular reporting deficiency include training cost valuation (selection, quantitation, and pricing of resources), number of participants, description of interventions (both primary and comparison) and setting, and discussion of generalizability. Abstracts contained, on average, fewer than half the desired details. Common methodological deficiencies include suboptimal choice of comparison interventions, failure to report incremental costs or combined expressions of cost and effectiveness, and absence of sensitivity analyses.

### Limitations

Most of the outcomes in this study required reviewer judgments of quality; to facilitate rigor and minimize subjectivity, we defined and applied specific operational criteria (Tab. S1 in ESM; Tab. 1). The CHEERS statement provides suggestions for additional details that we did not code (such as analytic methods, probability distributions, and base rates) because we viewed them as subordinate. Some included studies had cost as the primary focus, while for others costs were a secondary outcome; for the latter studies, reporting might have been appraised more favorably using reporting guidelines for other research designs (e.g., for randomized [9] and non-randomized [25] trials). The correlation between reporting and methodological quality likely reflects, in part, the fact that methods can only be judged from what is reported. Strengths of our study include the systematic search, duplicate review, use of a widely accepted reporting standard, and methodological appraisal using both cost-specific and general criteria.

### Integration with Prior Work

Two previous systematic reviews appraised cost evaluations in HPE, one using the BMJ Guidelines [15], the other focusing on cost ingredients [6], and both applying the MERSQI. Our review, which employed the more comprehensive CHEERS to appraise reporting and two additional approaches to appraise methodological quality, confirms those findings and identifies new areas of strength and deficiency. These findings also provide quantitative data elaborating the qualitative themes identified in our previous scoping review [19]. Our findings of significant deficiencies in reporting and methods parallel those from previous reviews of non-cost research in HPE [11, 14, 23, 26, 27]. Finally, looking outside HPE, in 2002 Clune rated the methodological quality of 56 cost evaluations as rhetoric (23 [41%]), and the remaining 33 as minimal (16 [48% of 33]), substantial (13 [39%]), and plausible (4 [12%]) [24]. Our findings roughly parallel his.

### Implications for education practice and research

Our findings have implications for research in CPD and for HPE research broadly. First, these data highlight specific gaps in reporting of CPD cost evaluations that warrant particular attention, such as valuation (resource selection, quantitation, and pricing), number trained, setting, description of the intervention and comparison, and discussions of generalizability (application in other training and clinical contexts). Unfortunately, poor reporting is ubiquitous, persistent, and refractory to remediation [28–32]. Indeed, our data show that reporting quality has improved only slightly since publication of the BMJ Guidelines [16] in 1996 and CHEERS [10] in 2013 (Tab. S3 in ESM). Clearly, the solution is not simple, and merely publishing reporting guidelines is insufficient. We call upon authors, editors, reviewers, and faculty development educators to collaborate to improve reporting quality.

Our findings also highlight specific deficiencies in titles and abstracts, and complement studies showing shortcomings of abstracts in other fields [14, 23, 33–35]. These deficiencies are not trivial: the abstract is critical in attracting consumers to read the full text, and essential to appropriate indexing for and searching of literature databases [36]; and titles are the “shortest possible abstract” [37]. Moreover, we found that structured abstracts are associated with more complete reporting. More informative and more structured abstracts have been requested for over 30 years [12, 13]. Tips for writing effective titles and abstracts are available [38, 39].

Our findings identify potential improvements in methodological quality. We emphasize the critical importance of resource quantitation, which is typically considered more important than actual costs [40–42], since prices vary widely by locale and over time, and quantities can be re-priced as needed using locally accurate values. Selection of relevant resources is also essential yet rarely reported and, we suspect, often done with little deliberate attention. Other potential methodological improvements include choosing an active comparison intervention, articulating the economic perspective, calculating combined expressions of cost and impact, formally analyzing incremental costs and cost-effectiveness, and performing sensitivity analyses.

Researchers and peer reviewers will benefit from our operational clarifications of MERSQI criteria for application to cost evaluations (Tab. 1) and our operational considerations for coding reporting quality with CHEERS (Tab. S1 in ESM), as they plan, report, and appraise such studies.

Finally, we note the overall paucity of cost-related studies. During the 63 years encompassed in this review, over 3520 original research studies were indexed under the MeSH term “Education, Medical, Continuing.” The 62 studies of CPD costs that we identified comprise only 1.8% of these (a proportion comparable to that found in previous reviews [6, 15]). We suggest that more studies that robustly measure and report the costs of CPD (and HPE generally) will contribute to informed decisions, efficient resource allocation, and ultimately a better-trained workforce.

## Supplementary Information

This material includes the full search strategy, operational definitions of the CHEERS elements, and a list of all included studies with key information
