# Supplementary material for: Quality of cost evaluations of physician continuous professional development: Systematic review of reporting and methods
Source: Perspect Med Educ. 2022 Mar 31;11(3):156–64. doi: 10.1007/s40037-022-00705-z (PMC9240125; doi:10.1007/s40037-022-00705-z)
Supplement: Supplementary file 1 — This material includes the full search strategy, operational definitions of the CHEERS elements, and a list of all included studies with key information [file 40037_2022_705_MOESM1_ESM.docx]

Supplemental Digital Appendices

#

# e-Box. Search strategy for studies of cost of continuing professional development

We used OVID to search the following databases, on April 23, 2020. We used the same search strategy for all databases.

- Ovid MEDLINE(R) and Epub Ahead of Print, In-Process & Other Non-Indexed Citations and Daily 1946 to April 22, 2020
- Embase 1974 to 2020 April 22,
- APA PsycInfo 1806 to April Week 2 2020,
- EBM Reviews - Cochrane Central Register of Controlled Trials March 2020,
- EBM Reviews - Cochrane Database of Systematic Reviews 2005 to April 22, 2020,

| # | Searches | Results |
| --- | --- | --- |
| 1 | "education medical continuing".hw. | 25414 |
| 2 | exp Education, Continuing/ | 93064 |
| 3 | ((continuing or continued or continuous or postgraduate) adj3 (health or medical or professional) adj3 (education or development)).ti,ab,hw,kw. | 52005 |
| 4 | 2 or 3 | 115168 |
| 5 | exp Physicians/ | 914857 |
| 6 | (Allergist* or Anaesthesiologist* or Andrologist* or Anesthesiologist* or cardiologist* or clinician* or dermatologist* or doctor* or endocrinologist* or Epidemiologist* or feldsher* or fellow or fellows or gastroenterologist* or gastrologist* or Geneticist* or geriatrician* or gerontologist* or Gynecologist* or Hematologist* or Hepatologist* or hospitalist* or Immunologist* or "Infectious Disease Specialist*" or "Internal Medicine Specialist*" or Internist* or medical or Microbiologist* or Neonatologist* or nephrologist* or neurologist* or Neurophysiologist* or neurosurgeon* or obstetrician* or oncologist* or Ophthalmologist* or Orthopedist* or otolaryngologist* or pathologist* or Pediatrician* or physician* or Physiologist* or podiatrist* or practitioner* or provider* or Psychiatrist* or Pulmonologist* or radiologist* or resident* or rheumatologist* or surgeon* or urologist*).ti,ab,hw,kw. | 8271061 |
| 7 | 5 or 6 | 8273613 |
| 8 | (4 and 7) or 1 | 72193 |
| 9 | exp *Economics, Medical/ or ("education medical continuing".hw. and exp Economics, Medical/) | 260969 |
| 10 | exp Cost-Benefit Analysis/ | 170873 |
| 11 | exp "cost effectiveness analysis"/ | 229704 |
| 12 | "Costs and Cost Analysis"/ | 119060 |
| 13 | exp "Cost Savings"/ | 79853 |
| 14 | ((economic* adj3 (benefit* or saving* or factor* or improv* or value)) or (value adj1 money) or budget* or "containing cost" or "containing costs" or "controlling cost" or "controlling costs" or "cost containment" or "cost control" or "cost effectiveness" or "cost saving*" or "Cost-Benefit" or "cost-benefits" or costs or efficiency or expenditure* or monetary or "return on investment" or revenue).ti,ab,hw,kw. | 2254760 |
| 15 | 9 or 10 or 11 or 12 or 13 or 14 | 2420662 |
| 16 | (referral adj3 (pattern* or increas* or improv*)).ti,ab,hw,kw. | 14222 |
| 17 | ((continuing or continued or continuous or postgraduate) adj3 (health or medical or professional) adj3 (education or development) adj5 (value or benefit or benefits or (improv* adj5 (care or outcome*)))).ti,ab,hw,kw. | 509 |
| 18 | 15 or 16 or 17 | 2433953 |
| 19 | 8 and 18 | 4584 |
| 20 | limit 19 to (dissertation abstract or editorial or erratum or note or addresses or autobiography or bibliography or biography or blogs or comment or dictionary or directory or interactive tutorial or interview or lectures or legal cases or legislation or news or newspaper article or overall or patient education handout or periodical index or portraits or published erratum or video-audio media or webcasts) [Limit not valid in PsycINFO,CCTR,CDSR,Embase,Ovid MEDLINE(R),Ovid MEDLINE(R) Daily Update,Ovid MEDLINE(R) In-Process,Ovid MEDLINE(R) Publisher; records were retained] | 279 |
| 21 | 19 not 20 | 4305 |
| 22 | remove duplicates from 21 | 3399 |

Further deduplication using a library protocol identified 46 duplicates, and then additional de-duplication in EndNote identified 15 additional duplicate articles, 1 full reprint, and 2 concurrently-published policy statements. These 64 articles were removed, leaving 3335 articles to be screened from this search strategy.

In addition, we found 3 articles from review of reference lists, for a total of 3338 articles screened.

# **Fig. S1** Trial flow for studies of cost of continuing professional development

Potentially relevant studies identified and screened for retrieval, after removal of duplicate publications (n=3338)

- 3335 from database search
- 3 from article reference lists

Studies excluded, with reasons (n=2748)

- 1375 Not original research
- 442 Not education of health professionals
- 401 No practicing physicians as learners
- 315 No cost or clinical outcomes
- 215 No comparison

Studies retrieved for more detailed evaluation (n=590)

Studies appropriate for inclusion in review (n=63)

Studies excluded, with reasons (n=527)

- 29 Not original research
- 35 Not education of health professionals
- 111 No practicing physicians as learners
- 282 No comparison of economic outcome
- 49 No evaluation of training costs
- 21 Meeting abstract

Studies included in review (n=62)

Studies excluded, with reasons (n=1)

- 1 Duplicate report of same study cohort

# **Table S1** Operational considerations for CHEERS elements coded in a systematic review of cost of continuing professional development

| **Element number** | **Element^a^** | **Elaboration^a^** | **Operational considerations** |
| --- | --- | --- | --- |
| 1 | TITLE AND ABSTRACT  Title | Identify the study as an economic evaluation or use more specific terms such as "cost-effectiveness analysis", and describe the interventions compared. | 1. Use of word "cost" or "economic" in title.  2. Includes 2 of: topic, audience (physician, CME), approach (instructional design, delivery format) |
| 2 | Abstract | Provide a structured summary of objectives, perspective, setting, methods (including study design and inputs), results (including base case and uncertainty analyses), and conclusions. | 1. Coded for none, no structure, structured (5 or fewer), highly structured (>5 headings)  2. Coded for presence of:   - Question/objective - Perspective (specific economic term) - Setting (explicit, using generalized terms ["tertiary-care hospital" not "St. Agnes Hospital"]) - Study design (either design for economic or non-economic is acceptable) - Participants (type ["physician"] + number) - Intervention: describe what was done with at least moderate detail (e.g., delivery modality, instructional methods, duration) - Comparison: as per Intervention, describe at least 1 - Inputs (training cost resources [ingredients]): at least partial listing of resources counted in cost analysis - Results (cost): Total or incremental cost of training for at least 1 intervention - Results (effectiveness): Total or change in non-training outcomes (details, not just P value) - Analysis of uncertainty or heterogeneity - Conclusions |
| 3 | INTRODUCTION  Background and objectives | Provide an explicit statement of the broader context for the study. Present the study question and its relevance for health policy or practice decisions. | 1. Describe need to study the cost/value of education (cost of clinical activities did not count)  2. Coded 4 elements of study question ("statement of study intent"): Population, Intervention, Comparison intervention, Outcome (cost and non-cost outcome separately) |
| 4 | METHODS  Target population and subgroups | Describe characteristics of the base case population and subgroups analysed, including why they were chosen. | Coded for physicians and patient separately: must report both eligibility (explicit statement of inclusion criteria) and number enrolled or providing data |
| 5 | Setting and location | State relevant aspects of the system(s) in which the decision(s) need(s) to be made. | Coded separately:  1. Training context: physical location of training, experience/qualifications of CME provider, or relationship between provider and physicians  2. Clinical context: patient demographics, or institution type, size, or infrastructure |
| 6 | Study perspective | Describe the perspective of the study and relate this to the costs being evaluated. | Must be explicit |
| 7 | Comparators | Describe the interventions or strategies being compared and state why they were chosen. | 1. Coded for primary intervention: Must include 3 details about the educational intervention, such as delivery modality, instructional strategy, duration. Topic does not count as a detail  2. Coded for 1 comparison intervention, if relevant: Same criteria |
| 8 | Time horizon | State the time horizon(s) over which costs and consequences are being evaluated and say why appropriate. | Must be explicit |
| 9 | Discount rate | Report the choice of discount rate(s) used for costs and outcomes and say why appropriate. | Must be explicit; also coded if not done with justification for not doing it |
| 10 | Choice of health outcomes | Describe what outcomes were used as the measure(s) of benefit in the evaluation and their relevance for the type of analysis performed. | Describe effectiveness (non-cost) outcomes |
| 11a | Measurement of effectiveness: Single study-based estimates | Describe fully the design features of the single effectiveness study and why the single study was a sufficient source of clinical effectiveness data. | Subjective coding of reporting of design features of effectiveness study; focused on reporting (ignored method quality) and considered only the effectiveness outcomes (ignored design of cost study). Rating options were: Excellent (near-complete description of data source, method, date, N, analysis); Good (some details missing; replication not possible); Poor (few details; unclear what was done) |
| 11b | Measurement of effectiveness: Synthesis-based estimates | Describe fully the methods used for identification of included studies and synthesis of clinical effectiveness data. | As per CHEERS |
| 12 | Measurement and valuation of  preference based outcomes | If applicable, describe the population and methods used to elicit preferences for outcomes. | Applicable only to preference-based outcomes (e.g., quality-adjusted life years) |
| 13a | Estimating resources and costs: Single study-based economic evaluation | Describe approaches used to estimate resource use associated with the alternative interventions. Describe primary or secondary research methods for valuing each resource item in terms of its unit cost. Describe any adjustments made to approximate to opportunity costs. | Valuation methods coded for explicit description of:  1. Selection of relevant resources (specific method or framework to identify most relevant resources)  2. Quantitation of resources used (vague, or specific method including: prospective log, retrospective document analysis, self-report by those trained, direct observation)  3. Pricing for each element (vague, or specific source including: log of actual costs/payments, estimate or percentage of total program budget, published/national fee schedule, shadow price) |
| 13b | Estimating resources and costs: Model-based economic evaluation | Describe approaches and data sources used to estimate resource use associated with model health states. Describe primary or secondary research methods for valuing each resource item in terms of its unit cost. Describe any adjustments made to approximate to opportunity costs. | As per CHEERS |
| 14 | Currency, price date, and  conversion | Report the dates of the estimated resource quantities and unit costs. Describe methods for adjusting estimated unit costs to the year of reported costs if necessary. Describe methods for converting costs into a common currency base and the exchange rate. | Must be explicit for both currency and year |
| 15 | Choice of model | Describe and give reasons for the specific type of decision-analytical model used. Providing a figure to show model structure is strongly recommended. | Model-based studies only; must be explicit |
| 16 | Assumptions | Describe all structural or other assumptions underpinning the decision-analytical model. | Model-based studies only; must be explicit |
| 17 | Analytical methods | Describe all analytical methods supporting the evaluation. This could include methods for dealing with skewed, missing, or censored data; extrapolation methods; methods for pooling data; approaches to validate or make adjustments (such as half cycle corrections) to a model; and methods for handling population heterogeneity and uncertainty. | Explicit description of methods used for statistical analysis of cost, cost-effectiveness, or net benefit (analysis non-cost outcomes was not considered) |
| 18 | RESULTS  Study parameters | Report the values, ranges, references, and, if used, probability distributions for all parameters. Report reasons or sources for distributions used to represent uncertainty where appropriate. Providing a table to show the input values is strongly recommended. | 1. Explicit listing of all resources counted in the cost analysis  2. Quantity of each resource used |
| 19 | Incremental costs and outcomes | For each intervention, report mean values for the main categories of estimated costs and outcomes of interest, as well as mean differences between the comparator groups. If applicable, report incremental cost-effectiveness ratios. | 1. Total cost for main intervention, and (if relevant) at least 1 comparison  2. Comparative (incremental) cost or cost-effectiveness for at least 1 intervention (e.g., incremental cost-effectiveness ratio)  3. Total non-cost outcome (effectiveness) for main intervention, and (if relevant) at least 1 comparison  4. Comparative effectiveness for at least 1 intervention (e.g., mean difference, risk ratio, odds ratio) |
| 20a | Characterising uncertainty: Single study-based economic evaluation | Describe the effects of sampling uncertainty for the estimated incremental cost and incremental effectiveness parameters, together with the impact of methodological assumptions (such as discount rate, study perspective). | Sensitivity analysis |
| 20b | Characterising uncertainty: Model-based economic evaluation | Describe the effects on the results of uncertainty for all input parameters, and uncertainty related to the structure of the model and assumptions. | Sensitivity analysis |
| 21 | Characterising heterogeneity | If applicable, report differences in costs, outcomes, or cost-effectiveness that can be explained by variations between subgroups of patients with different baseline characteristics or other observed variability in effects that are not reducible by more information. | Subgroup analysis |
| 22 | DISCUSSION  Study findings, limitations,  generalisability, and current  knowledge | Summarise key study findings and describe how they support the conclusions reached. Discuss limitations and the generalisability of the findings and how the findings fit with current knowledge. | 1. Summary of key study findings (succinct yet detailed & specific)  2. Summary of study limitations  3. Integration with prior related work  4. Consideration of generalizability (applicability to new context, such as potential differences in resource use, unit price, or clinical practice in different settings) |
| 23 | OTHER  Source of funding | Describe how the study was funded and the role of the funder in the identification, design, conduct, and reporting of the analysis. Describe other non-monetary sources of support. | Must be explicit |
| 24 | Conflicts of interest | Describe any potential for conflict of interest of study contributors in accordance with journal policy. | Must be explicit |

^a^ Element and elaboration are quoted from the CHEERS statement (Husereau, 2013).

# **Table S2** List of studies included in a systematic review of cost of continuing professional development

|  |  |  |  | **Reporting Index** | | | | | |  |  |  |  |  |
| --- | --- | --- | --- | --- | --- | --- | --- | --- | --- | --- | --- | --- | --- | --- |
| **Author (year)** | **Participants** | **No. physicians** | **Study design** | **Total** | **Title/ Abstr** | **Intro** | **Method** | **Result** | **Disc** | **Setting** | **Cost methods** | **Validity** | **Sensitivity** | **MERSQI** |
| Troutwine (1957)^1^ | Other/Vague | 478 | NR2 | 167 | 7 | 50 | 50 | 60 | 0 | Trn |  | Con |  | 8 |
| Stapleton (1973)^2^ | FM/IM | 102 | PP1 | 266 | 7 | 50 | 67 | 43 | 100 | Trn, Cln |  |  |  | 9 |
| McGehee (1974)^3^ | FM/IM, LabRad, OtherMed, Surg, Other/Vague | 525 | CS1 | 104 | 0 | 0 | 50 | 29 | 25 | Trn |  |  |  | 6 |
| Jewell (1978)^4^ | FM/IM | 16 | RCT | 171 | 0 | 50 | 71 | 50 | 0 | Trn |  |  |  | 13 |
| Walsh (1983)^5^ | All |  | PP1 | 185 | 20 | 50 | 40 | 25 | 50 | Trn, Cln | Quant, Price |  |  | 9.5 |
| Soumerai (1986)^6^ | Other/Vague | 435 | RCT | 367 | 33 | 100 | 71 | 63 | 100 | Trn, Cln | Quant |  |  | 12 |
| Landgren (1988)^7^ | All |  | NR2 | 254 | 20 | 50 | 71 | 63 | 50 | Trn |  |  |  | 11 |
| Rutz (1992)^8^ | FM/IM | 18 | PP1 | 376 | 47 | 100 | 83 | 71 | 75 | Cln | Price |  |  | 10.5 |
| Jorgens (1993)^9^ | FM/IM, [RN] |  | PP1 | 117 | 27 | 0 | 33 | 57 | 0 | Cln |  |  |  | 10.5 |
| Zimmerman (1994)^10^ | Other/Vague |  | NR2 | 277 | 20 | 50 | 57 | 50 | 100 | Trn, Cln | Price |  |  | 13 |
| Renneker (1995)^11^ | FM/IM, Surg | 7 | CS1 | 335 | 47 | 50 | 67 | 71 | 100 | Trn |  |  |  | 9 |
| Bruera (1997)^12^ | FM/IM, [NPPA] | 18 | CS1 | 168 | 33 | 0 | 67 | 43 | 25 | Trn, Cln |  |  |  | 6 |
| Digiusto (1998)^13^ | FM/IM | 34 | CS1 | 94 | 7 | 0 | 33 | 29 | 25 |  |  |  |  | 6 |
| Gomel (1998)^14^ | FM/IM | 628 | RCT | 425 | 53 | 100 | 71 | 100 | 100 | Trn, Cln | Quant, Price | IS | Sens | 14.5 |
| Morriss (1998)^15^ | FM/IM | 8 | PP1 | 347 | 53 | 50 | 83 | 86 | 75 | Cln |  |  |  | 12.5 |
| Reid (1998)^16^ | FM/IM | 269 | NR2 | 208 | 40 | 50 | 43 | 50 | 25 | Trn, Cln |  |  |  | 7 |
| Kaner (1999)^17^ | FM/IM | 128 | RCT | 319 | 60 | 50 | 71 | 63 | 75 | Trn |  |  |  | 12.5 |
| Appleby (2000)^18^ | FM/IM, [PG], [NPPA], [RN] | 62 | PP1 | 359 | 40 | 50 | 83 | 86 | 100 | Trn, Cln |  |  |  | 11 |
| McNulty (2000)^19^ | FM/IM | 101 | NR2 | 232 | 33 | 0 | 86 | 38 | 75 | Trn, Cln |  |  |  | 11 |
| Rossiter (2000)^20^ | FM/IM | 65 | NR2 | 281 | 47 | 50 | 71 | 63 | 50 | Cln |  |  |  | 13 |
| Kendrick (2001)^21^ | FM/IM |  | NR2 | 174 | 20 | 50 | 29 | 50 | 25 |  |  |  |  | 11 |
| Valori (2001)^22^ | FM/IM, [PG] | 123 | NR2 | 382 | 33 | 100 | 86 | 63 | 100 | Trn, Cln |  |  |  | 11 |
| Watson (2001)^23^ | FM/IM | 107 | RCT | 327 | 53 | 50 | 86 | 63 | 75 | Trn, Cln |  | IS | Sens | 16 |
| Robling (2002)^24^ | FM/IM | 123 | RCT | 255 | 60 | 50 | 57 | 63 | 25 |  |  |  |  | 12 |
| Allen (2003)^25^ | FM/IM | 10 | PP1 | 271 | 53 | 50 | 50 | 43 | 75 | Trn, Cln |  |  |  | 9.5 |
| Himpens (2003)^26^ | FM/IM, [PG] | 402 | PP1 | 145 | 20 | 50 | 0 | 75 | 0 |  |  |  |  | 6.5 |
| Lenow (2003)^27^ | FM/IM | 32 | PP1 | 185 | 20 | 0 | 83 | 57 | 25 | Trn, Cln | Quant, Price |  |  | 10.5 |
| Cohen (2004)^28^ | FM/IM | 20 | PP1 | 319 | 33 | 100 | 50 | 86 | 50 |  | Price |  |  | 11.5 |
| Taylor (2004)^29^ | FM/IM, [RN] | 98 | RCT | 358 | 60 | 50 | 86 | 63 | 100 | Cln | Price |  | Subgrp | 10.5 |
| Verstappen (2004)^30^ | FM/IM | 194 | RCT | 385 | 87 | 100 | 86 | 63 | 50 | Cln | Quant, Price | IS | Sens | 15 |
| Hogg (2005)^31^ | FM/IM | 106 | RCT | 403 | 53 | 100 | 100 | 50 | 100 | Trn, Cln | Quant, Price | Con, IS | Sens | 16 |
| Kiessling (2005)^32^ | FM/IM | 54 | RCT | 331 | 60 | 100 | 71 | 50 | 50 | Trn, Cln |  |  |  | 12 |
| Mahe (2005)^33^ | FM/IM, [NPPA], [RN] | 87 | PP1 | 251 | 33 | 50 | 50 | 43 | 75 | Cln |  |  |  | 10.5 |
| Shakespeare (2005)^34^ | OtherMed | 5 | PP1 | 278 | 40 | 50 | 67 | 71 | 50 | Trn |  |  |  | 9.5 |
| Simon (2005)^35^ | FM/IM, [NPPA] | 367 | RCT | 283 | 60 | 0 | 86 | 63 | 75 | Trn, Cln |  |  |  | 12 |
| Sullivan (2005)^36^ | Peds |  | RCT | 432 | 73 | 100 | 71 | 88 | 100 | Cln | Price |  | Sens | 13 |
| Claes (2006)^37^ | FM/IM |  | RCT | 411 | 67 | 100 | 57 | 88 | 100 |  | Price | Con, IS |  | 15 |
| Siriwardena (2007)^38^ | FM/IM, [RN] |  | PP1 | 301 | 47 | 50 | 83 | 71 | 50 | Trn, Cln | Price |  |  | 10.5 |
| Grassini (2008)^39^ | FM/IM | 133 | NR2 | 275 | 53 | 50 | 71 | 50 | 50 | Trn, Cln |  |  |  | 11 |
| Clausen (2009)^40^ | Peds, [NPPA] | 221 | PP1 | 307 | 33 | 50 | 67 | 57 | 100 | Trn |  |  |  | 6.5 |
| Foels (2009)^41^ | FM/IM | 310 | PP1 | 255 | 40 | 0 | 83 | 57 | 75 | Trn, Cln |  |  |  | 12.5 |
| Harris (2009)^42^ | FM/IM | 1869 | NR2 | 369 | 60 | 100 | 71 | 88 | 50 | Trn | Price |  |  | 9 |
| McKenna (2009)^43^ | FM/IM | 155 | RCT (mod) | 285 | 67 | 50 | 56 | 63 | 50 | Cln | Price |  |  | 11 |
| Sperl-Hillen (2010)^44^ | FM/IM | 41 | RCT | 392 | 67 | 100 | 100 | 50 | 75 | Trn, Cln | Sel |  |  | 14 |
| Walsh (2010)^45^ | All, [PG], [NPPA] |  | PP1 | 222 | 7 | 50 | 33 | 57 | 75 | Trn |  |  |  | 8.5 |
| Lopez-Picazo (2011)^46^ | FM/IM | 265 | RCT | 425 | 53 | 100 | 71 | 100 | 100 | Trn, Cln | Quant, Price |  |  | 13 |
| Qureshi (2011)^47^ | FM/IM | 61 | NR2 | 248 | 27 | 50 | 71 | 50 | 50 | Trn | Price |  |  | 9 |
| Schopf (2011)^48^ | FM/IM, [RN] | 46 | CS1 | 254 | 33 | 50 | 67 | 29 | 75 | Trn |  |  |  | 9.5 |
| Trogdon (2011)^49^ | FM/IM | 110 | NR2 (mod) | 399 | 60 | 100 | 89 | 75 | 75 | Trn | Sel | Con, IS |  | 15 |
| Butler (2012)^50^ | FM/IM | 263 | RCT | 373 | 73 | 50 | 100 | 75 | 75 | Trn, Cln | Sel, Quant, Price | Con |  | 14 |
| Devine (2012)^51^ | FM/IM |  | RCT (mod) | 351 | 87 | 0 | 89 | 75 | 100 | Trn | Sel, Price |  | Sens | 13 |
| Falcone (2012)^52^ | Surg |  | NR2 | 294 | 53 | 100 | 29 | 63 | 50 | Trn | Sel, Price |  |  | 11 |
| John (2014)^53^ | Peds, [RN] | 116 | PP1 | 281 | 47 | 50 | 67 | 43 | 75 | Trn, Cln |  |  |  | 10.5 |
| Nelson (2014)^54^ | FM/IM |  | NR2 (mod) | 308 | 60 | 50 | 63 | 60 | 75 | Cln | Sel, Quant, Price | Con, IS, RV | Sens | 13.5 |
| Nelson (2014)^55^ | FM/IM |  | PP1 (mod) | 320 | 60 | 50 | 75 | 60 | 75 | Trn, Cln | Sel, Quant, Price | Con, IS | Sens | 13.5 |
| Cantor (2015)^56^ | FM/IM, [Pharm], [NPPA], [RN] | 87 | RCT (mod) | 346 | 53 | 100 | 56 | 63 | 75 |  | Sel, Price | IS | Sens | 14 |
| Holuby (2015)^57^ | FM/IM, [Pharm], [RN] | 79 | NR2 | 249 | 40 | 0 | 71 | 63 | 75 | Trn |  |  |  | 11 |
| Pringle (2015)^58^ | EMed, Surg, [PG] | 18 | PP1 | 349 | 47 | 100 | 67 | 86 | 50 | Cln | Price |  |  | 10 |
| Vukovic (2015)^59^ | Anesth, FM/IM, LabRad, IntMedSp, Surg, [RN] | 71 | PP1 | 330 | 33 | 100 | 50 | 71 | 75 | Cln | Sel |  |  | 10.5 |
| Greenberg (2018)^60^ | Surg | 3 | CS1 | 270 | 47 | 0 | 67 | 57 | 100 | Trn |  |  |  | 9.5 |
| Jafar (2020)^61^ | FM/IM |  | RCT | 303 | 33 | 50 | 57 | 63 | 100 | Cln | Sel, Price | Con |  | 13 |
| Sloane (2020)^62^ | FM/IM, [NPPA] |  | NR2 | 350 | 60 | 50 | 83 | 57 | 100 | Trn, Cln | Price |  | Subgrp | 12 |

**Abbreviations**:

Participants: All are physicians in independent practice unless otherwise noted in [brackets]. All = all providers in institution or region, not otherwise specified; Anesth = anesthesiology; EMed = emergency medicine; FM/IM = family / internal / general medicine; IntMedSp = internal medicine subspecialty; LabRad = laboratory/pathology or radiology; OtherMed = other medical specialty; Peds = pediatrics or pediatric subspecialty; Surg = surgery; [PG] = physicians in postgraduate training; [Pharm] = pharmacist or pharmacy student; [NPPA] = nurse practitioner or physician assistant, or student; [RN] = nurse or nursing student.

Study design: RCT = randomized trial; NR2 = nonrandomized experiment (≥2 groups); PP1 = single-group pre/post-intervention or time series; CS1 = single group, single time point (cross-sectional); model = economic modeling study.

Setting: Trn = training context; Cln = clinical context.

Cost methods: Denotes the methods reported for cost estimation. Sel = selection; Quant = quantitation; Price = pricing.

Validity: Con = content evidence; IS = internal structure evidence; RV = relations with other variables evidence.

Sensitivity: Sens = sensitivity analysis; Subgrp = subgroup analysis.

MERSQI: Total quality appraisal score using the Medical Education Research Study Quality Instrument.

# **Table S3** Changes in reporting and methodological quality over time

|  | **Publication year** | | | |  |
| --- | --- | --- | --- | --- | --- |
| **Quality element** | **1957-1998**  **N=16** | **1999-2004**  **N=14** | **2005-2010**  **N=15** | **2011-2020**  **N=17** | **P value** |
| Overall reporting index, mean (SD) | 241 (105) | 285 (79) | 320 (66) | 321 (52) | 0.08 |
| Conflict of interest (reported yes or no), No. (%) | 0 (0%) | 1 (7%) | 11 (73%) | 12 (71%) | <0.001 |
| Quantitation, No. (%) | 3 (19%) | 2 (14%) | 1 (7%) | 4 (24%) | 0.70 |
| Pricing, No. (%) | 4 (25%) | 4 (29%) | 7 (47%) | 11 (65%) | 0.09 |
| Sensitivity analysis, No. (%) | 1 (6%) | 2 (14%) | 2 (13%) | 4 (24%) | 0.61 |
| Total MERSQI, mean (SD) | 9.8 (2.7) | 11.5 (2.3) | 11.4 (2.5) | 11.9 (1.9) | 0.15 |
| Subjective quality, mean (SD)* | 1.3 (0.6) | 1.4 (0.6) | 1.3 (0.6) | 1.7 (0.8) | 0.19 |

* Appraised using criteria defined by Clune (see main text for details) and scored as Plausible = 3, Substantial = 2, Minimal = 1.

# References

1. Troutwine CW. What are the real costs of postgraduate medical education. *Can Med Assoc J*. 1957;76:552-556.

2. Stapleton JF, Paullin AK. Hospital teaching conferences on home television. *Jama*. 1973;223:1131-1137.

3. McGehee EH, Clark JE, Coppola ED, Gonnella JS, Levit EJ. The Philadelphia County Medical Society self-evaluation examination. *Journal of Medical Education*. 1974;49:993-995.

4. Jewell SE. Students in CME. *Journal of Medical Education*. 1978;53:1008-1009.

5. Walsh PL. Cost avoidance in a regional continuing education center. *Mobius*. 1983;3:17-25.

6. Soumerai SB, Avorn J. Economic and policy analysis of university-based drug "detailing". *Medical Care*. 1986;24:313-331.

7. Landgren FT, Harvey KJ, Mashford ML, Moulds RF, Guthrie B, Hemming M. Changing antibiotic prescribing by educational marketing. *Medical Journal of Australia*. 1988;149:595-599.

8. Rutz W, Carlsson P, von Knorring L, Walinder J. Cost-benefit analysis of an educational program for general practitioners by the Swedish Committee for the Prevention and Treatment of Depression. *Acta Psychiatr Scand*. 1992;85:457-464.

9. Jorgens V. [Cost-benefit analysis of education programs and of treatment in type 2 diabetes]. *Diabete Metab*. 1993;19:510-513.

10. Zimmerman DR, Collins TM, Lipowski EE, Sainfort F. Evaluation of a DUR intervention: a case study of histamine antagonists. *Inquiry*. 1994;31:89-101.

11. Renneker M, Saner H. Low-cost flexible sigmoidoscopy screening: a community demonstration and education project. *J Cancer Educ*. 1995;10:25-30.

12. Bruera E, Selmser P, Pereira J, Brenneis C. Bus rounds for palliative care education in the community. *Cmaj*. 1997;157:729-732.

13. Digiusto EA, Leigh SV, Hardcastle DA, Currie JN. Effectiveness of CME workshops for alcohol and other drug-related interventions in general practice [2]. *Medical Journal of Australia*. 1998;169:116-117.

14. Gomel MK, Wutzke SE, Hardcastle DM, Lapsley H, Reznik RB. Cost-effectiveness of strategies to market and train primary health care physicians in brief intervention techniques for hazardous alcohol use. *Soc Sci Med*. 1998;47:203-211.

15. Morriss R, Gask L, Ronalds C, Downes-Grainger E, Thompson H, Leese B, et al. Cost-effectiveness of a new treatment for somatized mental disorder taught to GPs. *Family Practice*. 1998;15:119-125.

16. Reid DS, Weaver LE, Sargeant JM, Allen MJ, Mason WF, Klotz PJ, et al. Telemedicine in Nova Scotia: report of a pilot study. *Telemedicine Journal*. 1998;4:249-258.

17. Kaner EF, Lock CA, McAvoy BR, Heather N, Gilvarry E. A RCT of three training and support strategies to encourage implementation of screening and brief alcohol intervention by general practitioners. *British Journal of General Practice*. 1999;49:699-703.

18. Appleby L, Morriss R, Gask L, Roland M, Perry B, Lewis A, et al. An educational intervention for front-line health professionals in the assessment and management of suicidal patients (The STORM Project). *Psychol Med*. 2000;30:805-812.

19. McNulty CA, Kane A, Foy CJ, Sykes J, Saunders P, Cartwright KA. Primary care workshops can reduce and rationalize antibiotic prescribing. *J Antimicrob Chemother*. 2000;46:493-499.

20. Rossiter LF, Whitehurst-Cook MY, Small RE, Shasky C, Bovbjerg VE, Penberthy L, et al. The impact of disease management on outcomes and cost of care: a study of low-income asthma patients. *Inquiry*. 2000;37:188-202.

21. Kendrick T, Stevens L, Bryant A, Goddard J, Stevens A, Raftery J, et al. Hampshire depression project: changes in the process of care and cost consequences. *British Journal of General Practice*. 2001;51:911-913.

22. Valori RM, Brown CM, Strangeways P, Bradburn M. Reducing community dyspepsia drug costs: A controlled trial. *Gut*. 2001;49:495-501.

23. Watson M, Gunnell D, Peters T, Brookes S, Sharp D. Guidelines and educational outreach visits from community pharmacists to improve prescribing in general practice: a randomised controlled trial. *J Health Serv Res Policy*. 2001;6:207-213.

24. Robling MR, Houston HLA, Kinnersley P, Hourihan MD, Cohen DR, Hale J, et al. General practitioners' use of magnetic resonance imaging: an open randomized trial comparing telephone and written requests and an open randomized controlled trial of different methods of local guideline dissemination. *Clin Radiol*. 2002;57:402-407.

25. Allen M, Sargeant J, Mann K, Fleming M, Premi J. Videoconferencing for practice-based small-group continuing medical education: feasibility, acceptability, effectiveness, and cost. *Journal of Continuing Education in the Health Professions*. 2003;23:38-47.

26. Himpens B. The Pentalfa project. 2: profile of participants and economic aspects. *J Telemed Telecare*. 2003;9:104-108.

27. Lenow JL, Bales R, Smullens SN. The JeffCare preceptor model for asthma: a primary care physician tutorial training model. *Disease Management*. 2003;6:35-42.

28. Cohen D, Longo MF, Hood K, Edwards A, Elwyn G. Resource effects of training general practitioners in risk communication skills and shared decision making competences. *Journal of Evaluation in Clinical Practice*. 2004;10:439-445.

29. Taylor RS, Reeves BC, Ewings PE, Taylor RJ. Critical appraisal skills training for health care professionals: a randomized controlled trial [ISRCTN46272378]. *BMC Med Educ*. 2004;4:30.

30. Verstappen WHJM, Van Merode F, Grimshaw J, Dubois WI, Grol RPTM, Van der Weijden T. Comparing cost effects of two quality strategies to improve test ordering in primary care: A randomized trial. *International Journal for Quality in Health Care*. 2004;16:391-398.

31. Hogg W, Baskerville N, Lemelin J. Cost savings associated with improving appropriate and reducing inappropriate preventive care: cost-consequences analysis. *BMC Health Services Research*. 2005;5:20.

32. Kiessling A, Zethraeus N, Henriksson P. Cost of lipid lowering in patients with coronary artery disease by case method learning. *Int J Technol Assess Health Care*. 2005;21:180-186.

33. Mahe A, Faye O, N'Diaye HT, Konare HD, Coulibaly I, Keita S, et al. Integration of basic dermatological care into primary health care services in Mali. *Bulletin of the World Health Organization*. 2005;83:935-941.

34. Shakespeare TP, Mukherjee RK, Lu JJ, Lee KM, Back MF. Evaluation of an audit with feedback continuing medical education program for radiation oncologists. *J Cancer Educ*. 2005;20:216-221.

35. Simon SR, Majumdar SR, Prosser LA, Salem-Schatz S, Warner C, Kleinman K, et al. Group versus individual academic detailing to improve the use of antihypertensive medications in primary care: a cluster-randomized controlled trial. *American Journal of Medicine*. 2005;118:521-528.

36. Sullivan SD, Lee TA, Blough DK, Finkelstein JA, Lozano P, Inui TS, et al. A multisite randomized trial of the effects of physician education and organizational change in chronic asthma care: cost-effectiveness analysis of the Pediatric Asthma Care Patient Outcomes Research Team II (PAC-PORT II). *Arch Pediatr Adolesc Med*. 2005;159:428-434.

37. Claes N, Moeremans K, Frank B, Jef A, Jos V, Herman VL, et al. Estimating the cost-effectiveness of quality-improving interventions in oral anticoagulation management within general practice. *Value in Health*. 2006;9:369-376.

38. Siriwardena AN, Fairchild P, Gibson S, Sach T, Dewey M. Investigation of the effect of a countywide protected learning time scheme on prescribing rates of ramipril: interrupted time series study. *Family Practice*. 2007;24:26-33.

39. Grassini M, Verna C, Battaglia E, Niola P, Navino M, Bassotti G. Education improves colonoscopy appropriateness. *Gastrointestinal Endoscopy*. 2008;67:88-93.

40. Clausen MM, Armitage MD, Arnold RW. Overcoming barriers to pediatric visual acuity screening through education plus provision of materials. *J Aapos*. 2009;13:151-154.

41. Foels T, Hewner S. Integrating pay for performance with educational strategies to improve diabetes care. *Population Health Management*. 2009;12:121-129.

42. Harris JM, Jr., Novalis-Marine C, Amend RW, Surprenant ZJ. Promoting free online CME for intimate partner violence: what works at what cost? *Journal of Continuing Education in the Health Professions*. 2009;29:135-141.

43. McKenna C, Bojke L, Manca A, Adebajo A, Dickson J, Helliwell P, et al. Shoulder acute pain in primary health care: is retraining GPs effective? The SAPPHIRE randomized trial: a cost-effectiveness analysis. *Rheumatology (Oxford)*. 2009;48:558-563.

44. Sperl-Hillen JM, O'Connor PJ, Rush WA, Johnson PE, Gilmer T, Biltz G, et al. Simulated physician learning program improves glucose control in adults with diabetes. *Diabetes Care*. 2010;33:1727-1733.

45. Walsh K, Rutherford A, Richardson J, Moore P. NICE medical education modules: an analysis of cost-effectiveness. *Education for Primary Care*. 2010;21:396-398.

46. Lopez-Picazo JJ, Ruiz JC, Sanchez JF, Ariza A, Aguilera B. A randomized trial of the effectiveness and efficiency of interventions to reduce potential drug interactions in primary care. *Am J Med Qual*. 2011;26:145-153.

47. Qureshi NA, Neyaz Y, Khoja T, Magzoub MA, Haycox A, Walley T. Effectiveness of three interventions on primary care physicians' medication prescribing in Riyadh City, Saudi Arabia.[Erratum appears in East Mediterr Health J. 2011 Mar;17(3):249]. *Eastern Mediterranean Health Journal*. 2011;17:172-179.

48. Schopf T, Flytkjaer V. Doctors and nurses benefit from interprofessional online education in dermatology. *BMC Med Educ*. 2011;11:84.

49. Trogdon JG, Allaire BT, Egan BM, Lackland DT, Masters D. Training providers in hypertension guidelines: cost-effectiveness evaluation of a continuing medical education program in South Carolina. *Am Heart J*. 2011;162:786-793.e781.

50. Butler CC, Simpson SA, Dunstan F, Rollnick S, Cohen D, Gillespie D, et al. Effectiveness of multifaceted educational programme to reduce antibiotic dispensing in primary care: practice based randomised controlled trial. *Bmj*. 2012;344:d8173.

51. Devine A, Spencer A, Eldridge S, Norman R, Feder G. Cost-effectiveness of Identification and Referral to Improve Safety (IRIS), a domestic violence training and support programme for primary care: a modelling study based on a randomised controlled trial. *BMJ Open*. 2012;2:e001008.

52. Falcone JL, Watson AR. Surgical Morbidity and Mortality Conference using teleconferencing allows for increased faculty participation and moderation from satellite campuses and saves costs. *Journal of Surgical Education*. 2012;69:58-62.

53. John T, Morton M, Weissman M, O'Brien E, Hamburger E, Hancock Y, et al. Feasibility of a virtual learning collaborative to implement an obesity QI project in 29 pediatric practices. *International Journal for Quality in Health Care*. 2014;26:205-213.

54. Nelson SD, Nelson RE, Cannon GW, Lawrence P, Battistone MJ, Grotzke M, et al. Cost-effectiveness of training rural providers to identify and treat patients at risk for fragility fractures. *Osteoporosis International*. 2014;25:2701-2707.

55. Nelson RE, Battistone MJ, Ashworth WD, Barker AM, Grotzke M, Huhtala TA, et al. Cost effectiveness of training rural providers to perform joint injections. *Arthritis Care Res (Hoboken)*. 2014;66:559-566.

56. Cantor SB, Deshmukh AA, Luca NS, Nogueras-Gonzalez GM, Rajan T, Prokhorov AV. Cost-effectiveness analysis of smoking-cessation counseling training for physicians and pharmacists. *Addict Behav*. 2015;45:79-86.

57. Holuby RS, Pellegrin KL, Barbato A, Ciarleglio A. Recruitment of rural healthcare professionals for live continuing education. *Med*. 2015;20:28958.

58. Pringle K, Mackey JM, Modi P, Janeway H, Romero T, Meynard F, et al. "A short trauma course for physicians in a resource-limited setting: Is low-cost simulation effective?". *Injury*. 2015;46:1796-1800.

59. Vukovic M, Gvozdenovic BS, Rankovic M, McCormick BP, Vukovic DD, Gvozdenovic BD, et al. Can didactic continuing education improve clinical decision making and reduce cost of quality? Evidence from a case study. *Journal of Continuing Education in the Health Professions*. 2015;35:109-118.

60. Greenberg JA, Jolles S, Sullivan S, Quamme SP, Funk LM, Lidor AO, et al. A structured, extended training program to facilitate adoption of new techniques for practicing surgeons. *Surgical Endoscopy*. 2018;32:217-224.

61. Jafar TH, Gandhi M, de Silva HA, Jehan I, Naheed A, Finkelstein EA, et al. A Community-Based Intervention for Managing Hypertension in Rural South Asia. *New England Journal of Medicine*. 2020;382:717-726.

62. Sloane PD, Zimmerman S, Ward K, Kistler CE, Paone D, Weber DJ, et al. A 2-Year Pragmatic Trial of Antibiotic Stewardship in 27 Community Nursing Homes. *Journal of the American Geriatrics Society*. 2020;68:46-54.
